# A Graph-Space Optimal Transport Approach Based on Kaniadakis *κ*-Gaussian Distribution for Inverse Problems Related to Wave Propagation

**DOI:** 10.3390/e25070990

**Published:** 2023-06-28

**Authors:** Sérgio Luiz E. F. da Silva, João M. de Araújo, Erick de la Barra, Gilberto Corso

**Affiliations:** 1 Department of Applied Science and Technology, Politecnico di Torino, 10129 Torino, Italy; 2 Geoscience Institute, Fluminense Federal University, Niterói 24210-346, RJ, Brazil; 3 Department of Theoretical and Experimental Physics, Federal University of Rio Grande do Norte, Natal 59072-970, RN, Brazilgfcorso@gmail.com (G.C.); 4 School of Business, Universidad Católica del Norte, Coquimbo 1780000, CO, Chile

**Keywords:** *κ*-Gaussian distribution, optimal transport, seismic imaging, cycle skipping, non-linear optimization, Wasserstein metric, inverse problems, wave propagation

## Abstract

Data-centric inverse problems are a process of inferring physical attributes from indirect measurements. Full-waveform inversion (FWI) is a non-linear inverse problem that attempts to obtain a quantitative physical model by comparing the wave equation solution with observed data, optimizing an objective function. However, the FWI is strenuously dependent on a robust objective function, especially for dealing with cycle-skipping issues and non-Gaussian noises in the dataset. In this work, we present an objective function based on the Kaniadakis κ-Gaussian distribution and the optimal transport (OT) theory to mitigate non-Gaussian noise effects and phase ambiguity concerns that cause cycle skipping. We construct the κ-objective function using the probabilistic maximum likelihood procedure and include it within a well-posed version of the original OT formulation, known as the Kantorovich–Rubinstein metric. We represent the data in the graph space to satisfy the probability axioms required by the Kantorovich–Rubinstein framework. We call our proposal the κ-Graph-Space Optimal Transport FWI (κ-GSOT-FWI). The results suggest that the κ-GSOT-FWI is an effective procedure to circumvent the effects of non-Gaussian noise and cycle-skipping problems. They also show that the Kaniadakis κ-statistics significantly improve the FWI objective function convergence, resulting in higher-resolution models than classical techniques, especially when κ=0.6.

## 1. Introduction

The task of inferencing physical parameters from indirect observations arises in various practical problems. Determining parameters that cannot be directly observed remains a complex issue and involves a robust set of tools that compose the theoretical basis of the inverse problem theory [1]. The goal of an inverse problem consists of obtaining a quantitative model *m* that explains the observations (or observed data) by matching modeled data $d^{mod}=G\left(m\right)$ to observed data $d^{obs}$, in which *G* denotes the so-called forward operator. The forward operator maps the variables from the model space to the data space through a physical law [2]. For instance, we may want to determine the thermal diffusivity of a material (physical system) by analyzing the observed data: the temporal distribution of the diffusing material density at a determined location. In this regard, the model consists of the collective diffusion coefficient, and a diffusion equation represents the forward operator *G*. So, the diffusion coefficients are determined by optimizing an objective function, which measures the distance between modeled and observed data.

In this work, we consider a non-linear inverse problem that has attracted increasing interest in several fields, such as astrophysics [3], biomedicine [4], machine learning [5], and geophysics [6], named the Full-Waveform Inversion (FWI) [7]. FWI is a powerful imaging technique to obtain high-resolution quantitative physical models by analyzing the complete information of a collection of waveforms [8]. In particular, we consider the FWI technique in a geophysical context, in which the forward problem consists of simulating the propagation of acoustic waves by solving a wave equation. In this regard, the acoustic wave equation represents the forward operator *G*. At the same time, the data *d* and the model *m* are the pressure waveforms and the distribution of acoustic wave velocities (coefficients of the wave equation) of the subsurface medium. The inverse problem involves inferencing the coefficients of the wave equation (model parameters) by comparing the modeled data (wave equation solution) with the observed data by employing an objective function [9].

The objective function based on the least-squares method (or squared $l_{2}$-norm) is the most employed for handling FWI issues [7]. The least-squares objective function (from now on, classical objective function) computes the square root of the sum of the absolute squares of the residual data (or errors), the difference between the modeled and the observed data. Each objective function is closely connected to a statistical interpretation of the errors [2]. The classical objective function bears a relationship to Gaussian statistics. Indeed, in this classical framework, the errors are assumed to be independent and identically distributed according to a Gaussian probability distribution [10]. However, this assumption is sometimes adequate since the errors seldom are Gaussian in non-linear problems [11,12]. Let us remind the reader that errors arise from different natures, comprising the noise in the observations and uncertainties related to the physical rule employed in the forward problem. In fact, non-Gaussian noises are present in geophysical datasets and are caused by several elements, such as weather-related mechanisms [13] and instrument noise [14]. Several objective functions based on non-Gaussian statistics have been presented in the literature as alternative criteria. Non-Gaussian distributions exhibit much longer tails than the Gaussian ones, a crucial feature for dealing with erratic data (outliers) [15]. Several works have shown the effectiveness of non-Gaussian criteria in geophysical data inversions, such as objective functions based on Laplace distribution [16], Student’s t distribution [17], generalized approaches [18,19], and hybrid criteria [20,21].

Recently, ref. [22] introduced a new non-Gaussian criterion, namely, the κ-objective function, based on the Kaniadakis statistics (or κ-statistics) [23,24,25,26,27], which is robust to erratic data. The κ-objective function assumes that the errors are independent and identically distributed according to the κ-deformation of a Gaussian distribution (or κ-Gaussian distribution), in which the classical approach is a particular case [28]. The κ-Gaussian distribution arises from optimizing the Kaniadakis κ-entropy as a generalization of the well-known Gaussian distribution [29]. The κ-criterion exhibits robust characteristics thanks to the much longer tail of the κ-Gaussian distribution than the classical Gaussian probability function, which is crucial to mitigate the effects of non-Gaussian errors in FWI problems [30].

Due to the high computational efforts to solve the wave equation several times during the FWI process, the minimization process of the objective function is usually solved by local optimization methods [7]. Thus, FWI is prone to trapping into a non-informative local minimum if the initial background velocity model is not kinematically accurate [31]. Such an intrinsic limitation of FWI is associated with the absence of low-frequency contents, causing cycle-skipping issues [32,33]. Cycle-skipping is a phase ambiguity problem when the phase correspondence between two waveforms is greater than half a wavelength [34]. Although the approaches mentioned above are robust to non-Gaussian errors, they measure sample-by-sample the data misfit, making them sensitive to cycle skipping. Hence, a vast body of objective functions has been introduced for mitigating the cycle-skipping effects, such as those based on the waveform envelopes [35], convolutional filters [36], non-parametric techniques [37], and optimal transport metrics [38]; this is the methodology employed in the present study.

The theory of optimal transport (OT) was formally introduced by Gaspard Monge [39], who sought to understand the most effective allocation of resources by redistributing materials (mass) from sources to sinks. In recent years, OT theory has received much attention in broad literature (e.g., refs. [40,41,42,43]), such as geophysics issues [44,45,46]. However, the OT-based objective function is suitable for comparing probability distributions, that is, positive and normalized quantities, two requirements that seismic signals do not greet due to their oscillatory nature. In this way, waveforms are commonly distorted through transformations to satisfy the probability axioms, which may manufacture unwanted information. Indeed, several applications have demonstrated the effectiveness of OT-based objective functions to mitigate the effects of phase ambiguity; however, all assume that the errors obey Gaussian statistics.

In this work, we explore the κ-objective function in the context of OT theory to introduce an objective function resistant to non-Gaussian noise and less sensitive to cycle-skipping issues. In this regard, we propose a robust framework for matching seismic waveforms using the Wasserstein distance, a well-posed relaxation of the OT formulation. Furthermore, inspired by ref. [47], we consider the representation of the waveforms in the graph space suitable for real large-scale problems [33]. In this approach, the waveforms are represented by Dirac delta functions in a two-dimensional space (amplitude *versus* time).

We organize the present work as follows. In Section 2, we briefly introduce the theoretical basis of inverse problems in the context of κ-Gaussian statistics and their robustness properties. In Section 3, we present a well-posed relaxation of the original optimal transport formulation using the Kaniadakis κ-objective function. Then, in Section 4 we present FWI based on optimal transport and κ-Gaussian statistics in the context of the adjoint state method. In Section 5, we demonstrate how the proposed objective function deals with cycle-skipping issues and non-Gaussian noise by considering a Brazilian pre-salt case study. Finally, we devote Section 6 to the final remarks and future applications.

## 2. Inverse Problems in the Context of Kaniadakis κ-Statistics

In science issues, several practical problems are data-centric. Indeed, determining a quantitative physical model that explains the observations is crucial to more accurately model and describe a wide variety of existing physical systems. In this context, the inverse problem theory is an excellent tool.

From a practical point of view, an inverse problem is formulated as an optimization task for obtaining a quantitative model by comparing modeled data to observed data. Modeled data are calculated using an appropriate physical law. The comparison between modeled and observed data is performed through an objective function. In the classical approach, the objective function is constructed from the assumption that the errors (the difference between modeled and observed data) obey Gaussian statistics. Let $\vec{\varepsilon }=\left\{\varepsilon_{1},\varepsilon_{2},\cdots ,\varepsilon_{N}\right\}$ be the errors. From the assumption that the errors are independent and identically distributed according to a standard Gaussian distribution,

(1) $$p_{0}\left(\varepsilon_{i}\right)=\frac{1}{\sqrt{2\pi }}\exp\left(-\frac{1}{2}\varepsilon_{i}^{2}\right),$$

we can determine the associated likelihood function as follows [11]:

(2) $$L_{0}=\prod_{i=1}^{N}p_{0}\left(\varepsilon_{i}\right)={\left(\frac{1}{\sqrt{2\pi }}\right)}^{N}\exp\left(-\frac{1}{2}\sum_{i=1}^{N}\varepsilon_{i}^{2}\right),$$

where $L_{0}$ is the Gaussian likelihood. The use of index 0 will become clear later on. It is worth remembering that the standard Gaussian distribution can be determined from the maximization of the Boltzmann–Gibbs–Shannon entropy subject to the normalization condition

(3) $$\int_{-\infty }^{+\infty }p\left(\varepsilon \right)d\varepsilon =1$$

and the unit variance constraint,

(4) $$\int_{-\infty }^{+\infty }\varepsilon^{2}\;p\left(\varepsilon \right)d\varepsilon =1.$$

In inverse problems, the errors $\vec{\varepsilon }$ depend on the model parameter *m* and are computed through the difference between modeled ($d^{mod}=G\left(m\right)$) and observed ($d^{obs}$) data, i.e., $\varepsilon_{i}\left(m\right)=d_{i}^{mod}\left(m\right)-d_{i}^{obs}$, where *G* represents the forward operator. In this way, obtaining the model parameter can be performed by employing the maximum likelihood estimation (MLE) method, which is achieved by maximizing the likelihood function as follows:

(5) $$\hat{m}=\max_{m}L_{0}\left(m|d^{obs}\right)$$

where $\hat{m}$ represents the estimated model. The MLE estimates an unknown model parameter by considering that its optimal value maximizes the probability that the observed data are measured. Since the minimum of the negative log-likelihood coincides with the maximum of the likelihood function, maximizing $L_{0}$ (5) is equivalent to minimizing the negative log-likelihood, i.e.,

(6) $$\max_{m}L_{0}\left(m|d^{obs}\right)\equiv \min_{m}-\ln\left(L_{0}\left(m|d^{obs}\right)\right).$$

From the principle of maximum likelihood, an objective function $\phi_{0}$ can be obtained from [11]:

(7) $$\phi_{0}\left(m\right)\propto -\ln\left(L_{0}\left(m|d^{obs}\right)\right),$$

which can be rewritten as:

(8) $$\phi_{0}\left(m\right)\propto \frac{N}{2}\ln\left(2\;\pi \right)+\frac{1}{2}\sum_{i=1}^{N}\varepsilon_{i}^{2}$$

(9) $$\phi_{0}\left(m\right)=\frac{1}{2}\sum_{i=1}^{N}\varepsilon_{i}^{2}.$$

We notice that minimizing Equation (8) or (9) is the same since the term $\frac{N}{2}\ln\left(2\;\pi \right)$ is constant. The latter equation is well-known and used in solving problems via the least squares method. Please see Section 2 of ref. [48] for more detail.

However, due to the non-Gaussianity of the errors, it is reasonable to assume that the errors are non-Gaussian. In this study, we consider that the errors are distributed according to a Kaniadakis κ-Gaussian distribution of the form [22]:

(10) $$p_{\kappa }\left(\varepsilon_{i}\right)=\frac{1}{Z_{\kappa }}\exp_{\kappa }\left(-\beta_{\kappa }\;\varepsilon_{i}^{2}\right),$$

where $Z_{\kappa }$ is a normalizing constant, $\beta_{\kappa }$ is a scale parameter, and 

(11) $$\exp_{\kappa }\left(y\right)=\exp\left(\frac{1}{\kappa }\mathrm{arcsinh}\left(\kappa y\right)\right)={\left(\sqrt{1+\kappa^{2}y^{2}}+\kappa y\right)}^{\frac{1}{\kappa }}$$

with 0≤|κ|<1, is the κ-exponential function [26], a generalization of the exponential function. The κ-exponential becomes the ordinary exponential function in the limit κ→0: $\exp_{0}\left(y\right)=\exp\left(y\right)$.

**Figure 1:**
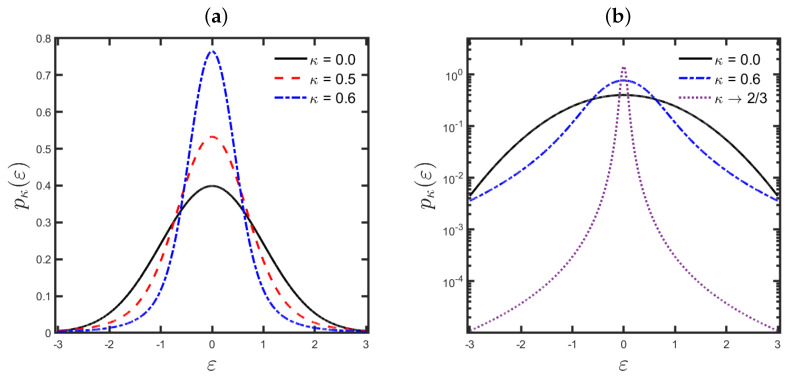
Probability plots of the κ-Gaussian distribution (10) for some κ-values using (**a**) a linear scale and (**b**) a linear scale on the axis of ordinates, and a logarithmic scale on the axis of abscissas. The solid black line represents the standard Gaussian distribution (κ→0).

Considering the normalization (3) and unitary variance (4) conditions, we obtain

(12) $$Z_{\kappa }=\sqrt{\frac{\pi }{\beta_{\kappa }}}\frac{{\left|2\kappa \right|}^{-1/2}}{1+\frac{1}{2}\left|\kappa \right|}\frac{\Gamma \left(\frac{1}{\left|2\kappa \right|}-\frac{1}{4}\right)}{\Gamma \left(\frac{1}{\left|2\kappa \right|}+\frac{1}{4}\right)}$$

and

(13) $$\beta_{\kappa }=\frac{{\left|2\kappa \right|}^{-1}}{2}\frac{1+\frac{1}{2}\left|\kappa \right|}{1+\frac{3}{2}\left|\kappa \right|}\frac{\Gamma \left(\frac{1}{\left|2\kappa \right|}-\frac{3}{4}\right)\;\Gamma \left(\frac{1}{\left|2\kappa \right|}+\frac{1}{4}\right)}{\Gamma \left(\frac{1}{\left|2\kappa \right|}+\frac{3}{4}\right)\;\Gamma \left(\frac{1}{\left|2\kappa \right|}-\frac{1}{4}\right)}$$

holding for |κ|<2/3. The standard Gaussian distribution (1) is a particular case of the κ-Gaussian distribution (10) in the classical limit κ→0 since $\lim_{\kappa \to 0}\beta_{\kappa }=\frac{1}{2}$ and $\lim_{\kappa \to 0}Z_{\kappa }=\sqrt{2\pi }$. Figure 1 depicts the plots of the κ-Gaussian distribution (10) for typical κ-values, with the solid black curve referring to the standard Gaussian distribution (κ→0).

Because we assume that the errors are independent and identically distributed by the power law distribution represented in (10), we can calculate the corresponding objective function by estimating the most likely state using the probabilistic maximum likelihood method:

(14) $$\underset{m}{min}\;\;\;\phi_{\kappa }\left(m\right)\equiv \max_{m}L_{\kappa }\left(m|d^{obs}\right),$$

where $L_{\kappa }\left(m|d^{obs}\right):=\prod_{i=1}^{N}p_{\kappa }\left(\varepsilon_{i}\left(m\right)\right)$ represents the likelihood function. It is crucial to remember that minimizing the negative log-likelihood is the same as maximizing the likelihood function. In this way, the objective function $\phi_{\kappa }$ can be obtained from (14):

(15) $$\phi_{\kappa }\left(m\right)\propto N\ln\left(Z_{\kappa }\right)-\sum_{i=1}^{N}\ln\left[\exp_{\kappa }\left(-\beta_{\kappa }\varepsilon_{i}^{2}\left(m\right)\right)\right]$$

(16) $$\phi_{\kappa }\left(m\right)=-\sum_{i=1}^{N}\ln\left[\exp_{\kappa }\left(-\beta_{\kappa }\varepsilon_{i}^{2}\left(m\right)\right)\right],$$

where $\phi_{\kappa }$ is the κ-objective function, which converges to the classical objective function (9) in the limit κ→0.

**Figure 2:**
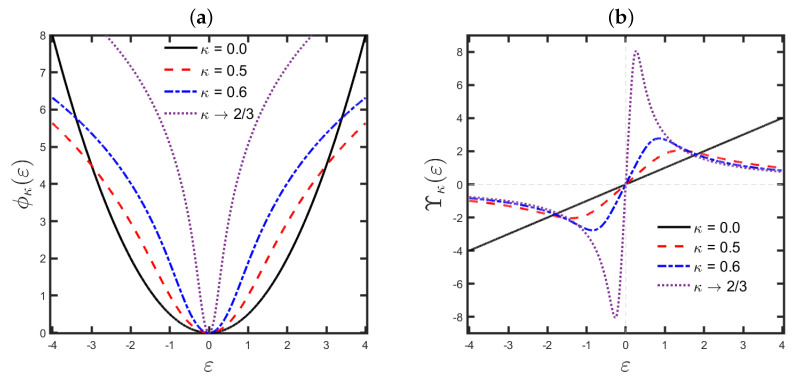
(**a**) Graphical representation of the κ-objective function (16), and (**b**) the associated influence function (18) for some κ-values. The solid black line represents the classical criterion (κ→0).

The κ-objective function is not easily influenced by aberrant measurements (outliers), as it is based on κ-Gaussian criteria [49]. To demonstrate this, we compute the influence function Υ related to the objective function. According to ref. [50], a statistical criterion is not robust if Υ→±∞ under |ε|→∞, and robust (outlier-resistant) if Υ→0 under ε→±∞. Given a model $m_{i}$, the influence function is defined by [50]:

(17) $$\Upsilon_{\kappa }\left(m_{i}\right):=\frac{\partial \phi_{\kappa }\left(\varepsilon |m_{i}\right)}{\partial \varepsilon },$$

where $\phi_{\kappa }\left(\varepsilon |m_{i}\right)$ is the κ-objective function computed from the errors ε given the model $m_{i}$. Thus, the κ-objective function generates the following influence function:

(18) $$\Upsilon_{\kappa }=\frac{2\beta_{\kappa }\varepsilon }{\sqrt{1+\kappa^{2}\beta_{\kappa }^{2}\varepsilon^{4}}}$$

for 0<κ<2/3, with $\Upsilon_{\kappa }=\Upsilon_{\kappa }\left(m_{i}\right)$ and $\varepsilon =\varepsilon (m_{i})$. We notice that, as ε tends to ±∞, the influence function associated with the κ-criterion ($\Upsilon_{\kappa }$) approaches to 0; the κ-objective function is then robust (outlier-resistant). Indeed, the influence function in its valid domain (0<κ<2/3) is proportional to 1/ε for large errors (suppressing these) and to ε for small errors (magnifying these). Figure 2 depicts the behavior of the κ-objective function and the associated influence function.

## 3. Optimal Transport Metric Based on Kaniadakis κ-Statistics

In 1781, Gaspard Monge first raised a challenger transportation problem [39], which consisted of moving a pile of sand from one place to another optimally and without losing mass. As formulated by Monge, the optimal transport (OT) issue is an ill-posed problem; hence the solution, if it exists, is not unique. Nearly 200 years later, Leonid Kantorovitch proposed a well-posed relaxation of Monge’s OT problem in the context of optimal economic resource allocation  [51]. In this regard, Kantorovich proposed what is now known as the Kantorovich–Rubinstein metric (also referred to as Wasserstein distance), which earned him the 1975 Nobel Memorial Prize in Economic Science.

The Wasserstein criterion is a metric that defines a distance between two probability distributions. Let us consider two sets of points $\Omega_{1}=\left\{x_{i};i=1,2,\cdots ,N_{1}\right\}$ and $\Omega_{2}=\left\{y_{j};j=1,2,\cdots ,N_{2}\right\}$, in which each point $x_{i}$ and $y_{j}$ are represented by “mass” functions, namely $\mu (x_{i})$ and $\upsilon (y_{j})$, respectively. Considering the mass conservation constraint ($\sum_{i}\mu \left(x_{i}\right)=\sum_{j}\upsilon \left(y_{j}\right)=1$), we can define the κ-optimal total transport cost $W_{\kappa }$ as  [30,40]:

(19) $$W_{\kappa }\left(\mu ,\upsilon \right)=\underset{T\in \Lambda (\mu ,\upsilon )}{min}\;\sum_{i,j}T_{i,j}\;\phi_{\kappa ,i,j},$$

where Λ(μ,υ) denotes the set of transport maps T defined in

(20) $$\Lambda \left(\mu ,\upsilon \right)=\left\{T_{i,j}\geq 0,\;\forall \left(i,j\right);\;\sum_{j=1}^{N_{2}}T_{i,j}=\mu \left(x_{i}\right),\;\forall \;\;i;\;\sum_{i=1}^{N_{1}}T_{i,j}=\upsilon \left(y_{j}\right),\;\forall \;\;j\right\}.$$

The transport map T assigns how many “sand particles” from $\mu (x_{i})$ should be transported to $\upsilon (y_{j})$ for each pair $\left(x_{i},y_{j}\right)$, while the κ-objective function maps each pair $\left(x_{i},y_{j}\right)$ to [0;+∞]. The Monge–Kantorovich transportation relaxed problem (19), using the Kaniadakis κ-statistics, can therefore be solved by determining an optimal transport plan T that minimizes the κ-optimal total transport cost $W_{\kappa }$ from μ to υ, given an κ-objective function $\phi_{\kappa }$.

Let us consider a metric space P(X×Y) formed by a set of probability measures, in which X and Y are two separable and complete metric spaces with μ∈X and υ∈Y. From this point forward, for practical reasons, we assume that $X=Y\subset R^{N}$ (with $N_{1}=N_{2}=N\in N$). In addition, let us consider the mass distributions μ and υ represented in terms of the Dirac delta function as follows: $\mu \left(x\right)=\frac{1}{N}\sum_{l=1}^{N}\delta \left(x-u_{l}\right)$ and $\upsilon \left(y\right)=\frac{1}{N}\sum_{l=1}^{N}\delta \left(y-w_{l}\right)$, in which $u_{l}\in \Omega_{1}$ and $w_{l}\in \Omega_{2}$ point out the data points describing μ(x) and υ(x). In this context, we can reformulate the optimization problem in Equation (19) as follows:

(21) $$W_{\kappa }\left(\mu ,\upsilon \right)=\underset{T_{i,j}}{min}\;-\frac{1}{N}\sum_{i,j=1}^{N}T_{i,j}\;\;\ln\left\{\exp_{\kappa }\left[-\beta_{\kappa }{\left(\mu \left(x_{i}\right)-\upsilon \left(y_{j}\right)\right)}^{2}\right]\right\}$$

subject to

(22) $$T_{i,j}\geq 0,\;\sum_{j=1}^{N}T_{i,j}=1,\;\sum_{i=1}^{N}T_{i,j}=1.$$

From a practical viewpoint, we notice that solving (21) consists of obtaining an optimal transport plan that links data points from P(X) to the corresponding data points in P(Y) that minimizes the κ-optimal total transport cost $W_{\kappa }$. Although each element of the optimal transport plan $T_{i,j}$ can assume fractional values, a classic result states that the optimal solution values are integer values, specifically 0 or 1 when the constraints described in Equation (22) are considered [52,53]. Indeed, obtaining the minimum of $W_{\kappa }$ implicates solving a combinatorial optimization issue, which can be defined as:

(23) $$W_{\kappa }\left(\mu ,\upsilon \right)=\underset{\sigma \in S}{min}\;-\frac{1}{N}\sum_{i=1}^{N}\ln\left\{\exp_{\kappa }\left[-\beta_{\kappa }{\left(\mu \left(x_{\sigma (i)}\right)-\upsilon \left(y_{i}\right)\right)}^{2}\right]\right\},$$

where σ represents a permutation solution for the linear sum assignment problem in (21) related with T, and S(N)={1,2,···,N} is a set of permutations. Equation (23) represents the Wasserstein metric in the context of κ-Gaussian statistics.

Naturally, the Wasserstein metric based on Kaniadakis κ-statistics appreciates the advantages provided by κ-Gaussian statistics. However, this approach in this format is only valid for comparing probability distributions, which is not interesting for geophysical applications like the FWI case. This incompatibility is because seismic signals are not normalized and positive-definite quantities like probability functions.

## 4. Kaniadakis κ-Graph-Space Optimal Transport FWI

### 4.1. FWI Based on Kaniadakis κ-Gaussian Distribution

In this section, we present the main elements of FWI based on the Kaniadakis κ-Gaussian distribution, the metric explained in Section 2. The FWI is a non-linear inverse problem whose main goal consists of inferring a quantitative physical model by comparing modeled waveforms (modeled data) with measured waveforms (observed data) [7]. FWI is often formulated as a gradient-based minimization due to the computational costs, in which the model parameters are iteratively updated, from an initial model $m_{0}$, as follows [8]:

(24) $$m_{i+1}=m_{i}-\alpha_{i}\;h_{\kappa }\left(m_{i}\right)\;\mathrm{for}\;\;i=0,1,2,\cdots ,N_{iter},$$

where *m* represents the model parameter, $\alpha_{i}>0$ is the so-called step length [54], $N_{iter}$ represents the number of FWI iterations, and $h_{\kappa }$ denotes the descent direction at the *i*-th iteration.

In this work, we employ a non-linear conjugate gradient optimization method based on the so-called Polak–Ribière–Polyak algorithm. In this regard, the descent direction is defined by [55,56]:

(25) $$h_{\kappa }\left(m_{i}\right)=\left\{\begin{array}{c} \;\nabla_{m}\phi_{\kappa }\left(m_{0}\right)\;,\;\;\mathrm{if}\;\;i=0\\ \nabla_{m}\phi_{\kappa }\left(m_{i}\right)+\zeta_{\kappa }\left(m_{i}\right)h_{\kappa }\left(m_{i-1}\right),\;\;\mathrm{for}\;\;i=1,2,\cdots ,N_{iter} \end{array}\right.$$

with

(26) $$\zeta_{\kappa }\left(m_{i}\right)=\frac{\nabla_{m}\phi_{\kappa }\left(m_{i}\right)\left(\nabla_{m}\phi_{\kappa }\left(m_{i}\right)-\nabla_{m}\phi_{\kappa }\left(m_{i-1}\right)\right)}{\nabla_{m}\phi_{\kappa }\left(m_{i-1}\right)\nabla_{m}\phi_{\kappa }\left(m_{i-1}\right)},$$

where $\nabla_{m}\phi_{\kappa }\left(m\right)$ is the gradient of the κ-objective function.

Thus, it is remarkable that the objective function plays a crucial role in obtaining models via FWI, which is defined for our problem as (10):

(27) $$\underset{m}{min}\;\phi_{\kappa }\left(m\right):=-\sum_{s,r}\int_{0}^{T}\ln\left\{\exp_{\kappa }\left[-\beta_{\kappa }\;{\left(\Gamma_{s,r}\psi_{s}\left(\vec{x},m,t\right)-d_{s,r}\left({\vec{x}}_{s,r},t\right)\right)}^{2}\right]\right\}dt,$$

where $\Gamma_{s,r}\psi_{s}=d_{s,r}^{mod}$ and $d_{s,r}=d_{s,r}^{obs}$ represent the modeled and observed data generated by the seismic source *s* and recorded in the receiver *r*, while $\vec{x}\in R^{2}$ and t∈[0,T] denote the spatial coordinates and the seismic acquisition time.

It is worth mentioning that the observed data $d_{s,r}$ are registered only in the receiver positions $\vec{x}={\vec{x}}_{s,r}$, the available and chosen positions during a seismic survey. The seismic wavefield $\psi_{s}$ is computed in the entire physical domain for each seismic source *s* by solving a wave equation. Thus, $\Gamma_{s,r}$ represents a sampling operator that acts as a measurement processor onto the receiver *r* from the source *s*. In this work, we consider the acoustic case; therefore, $\psi_{s}$ are the pressure wavefields that satisfy the following model:

(28) $$\frac{1}{c^{2}\left(\vec{x}\right)}\frac{\partial^{2}\psi_{s}\left(\vec{x},t\right)}{\partial t^{2}}-\nabla^{2}\psi_{s}\left(\vec{x},t\right)=g_{s}\left(t\right)\delta \left(\vec{x}-{\vec{x}}_{s}\right)$$

where $g_{s}$ represents a seismic source signature at the fixed position $\vec{x}={\vec{x}}_{s}$, *c* is the *P*-wave velocity model of the medium, and $\nabla^{2}$ denotes de Laplacian operator.

Thus, the gradient of the κ-objective function (27) with respect to the model parameters is given by:

(29) $$\nabla_{m}\phi_{\kappa }\left(m\right)=\frac{\partial \phi_{\kappa }\left(m\right)}{\partial m_{l}}=2\beta_{\kappa }\;\sum_{s,r}\int_{0}^{T}\frac{J_{s,r}\left(m,t\right)\Delta d_{s,r}\left(m,t\right)}{\sqrt{1+\kappa^{2}\beta_{\kappa }^{2}\Delta d_{s,r}^{4}\left(m,t\right)}}dt,$$

where $\Delta d_{s,r}\left(m,t\right)=\Gamma_{s,r}\psi_{s}\left(\vec{x},m,t\right)-d_{s,r}\left({\vec{x}}_{s,r},t\right)$ represents the error (or residual data) and

(30) $$J_{s,r}\left(m,t\right)=\frac{\partial }{\partial m_{l}}\left(\Gamma_{s,r}\psi_{s}\left(\vec{x},m,t\right)\right)$$

is known as the Fréchet derivative. It is worth emphasizing that FWI problems involve many elements from the model parameters that typically comprise $10^{6}$ to $10^{12}$ variables (coefficients of the wave equation). In this context, we need to solve the wave equation once in the forward modeling process plus at least $10^{6}$ times in calculating the gradient of the κ-objective function through Fréchet derivatives, being unfeasible in industrial problems.

### 4.2. Adjoint-State Method

Since calculating Fréchet derivatives can be computationally prohibitive, we compute the gradient of the κ-objective function using the adjoint-state method, which was developed in the 1970s [57]. There are several ways to formulate the state-adjoint approach, such as in techniques based on the augmented Lagrangian method or Green’s functions. However, in this work we consider the perturbation theory to calculate the gradient efficiently. We notice that the κ-objective function can be written as:

(31) $$\phi_{\kappa }\left(m\right)=f\left(\psi (m),m\right),$$

where ψ is a state variable that belongs to the complex space Q; ψ satisfies the following equation of state:

(32) $$F\left(\psi (m),m\right)=A\left(m,t\right)\psi \left(m,t\right)-g\left(t\right)=0,$$

in which we suppress the subscript *s* for the sake of a simplified notation. In the latter equation, A(m,t)ψ(t)=q(t) represents the wave equation written in a compact form, where $A\left(m,t\right)=m\frac{\partial^{2}}{\partial t^{2}}-\nabla^{2}$ is the d’Alembert wave operator with $m=\frac{1}{c^{2}\left(\vec{x}\right)}$ belonging to the real space M, whilst $g\left(t\right)=g_{s}\left(t\right)\delta \left(\vec{x}-{\vec{x}}_{s}\right)$.

Suppose we consider an arbitrary variation δm concerning the model parameter *m*. In that case, the state variable ψ will be disturbed by a variation δψ; consequently, the κ-objective function in Equation (31) will also be disturbed. In this way, we have to:

(33) $$\delta \phi_{\kappa }=\frac{\partial f(\psi ,m)}{\partial m}\delta m+{\langle \frac{\partial f(\psi ,m)}{\partial \psi_{j}},\delta \psi \rangle }_{Q},$$

where we only consider the first-order terms in δm and δψ. Furthermore, $\psi_{j}$ is any element of the space Q, and ${\langle ,\rangle }_{Q}$ is the inner product in Q.

It is worth emphasizing that the perturbations δm and δψ also induce variations in the equation of state (32). Moreover, assuming that there is a unique solution ψ for any model parameter *m*, we can state that ψ+δψ is the unique solution of F(ψ+δψ,m+δm)=0. In other words, for a physical realization ψ (that is, F(ψ,m)=0), we have the following first-order development in δm and δψ:

(34) $$F\left(\psi +\delta \psi ,m+\delta m\right)=F\left(\psi ,m\right)+\left(\frac{\partial F(\psi ,m)}{\partial m}\right)\delta m+\left(\frac{\partial F(\psi ,m)}{\partial \psi_{j}}\right)\delta \psi =0.$$

From the latter equation, we have that the perturbation in the state variable ψ is given by:

(35) $$\delta \psi =-{\left(\frac{\partial F(\psi ,m)}{\partial \psi_{j}}\right)}^{-1}\left(\frac{\partial F(\psi ,m)}{\partial m}\right)\delta m,$$

where $a^{-1}$ denotes the inverse of *a*. So, replacing the resulting from Equation (35) in Equation (33), we have an efficient way to compute the gradient of the κ-objective function without the Fréchet derivatives:

(36) $$\delta \phi_{\kappa }=\frac{\partial f(\psi ,m)}{\partial m}\delta m-{\langle \frac{\partial f(\psi ,m)}{\partial \psi_{j}},{\left(\frac{\partial F(\psi ,m)}{\partial \psi_{j}}\right)}^{-1}\left(\frac{\partial F(\psi ,m)}{\partial m}\right)\delta m\rangle }_{Q}.$$

On the other hand, to obtain an intuitive way to calculate the gradient, Equation (36) can be rewritten so that in the inner product in Q, one of the terms varies only with ψ and the other with *m*. For this, we consider the following adjoint operator property for any *x* and *y* variables:

(37) $$\langle x,Ry\rangle =\langle R^{†}x,y\rangle$$

where $R^{†}$ is the adjoint operator of R, while the superscript † represents the adjoint operation (complex-conjugate transpose). Applying the property (37) to the second term of Equation (36), we obtain:

(38) $$\delta \phi_{\kappa }=\frac{\partial f(\psi ,m)}{\partial m}\delta m-{\langle {\left[{\left(\frac{\partial F(\psi ,m)}{\partial \psi_{j}}\right)}^{-1}\right]}^{†}\frac{\partial f(\psi ,m)}{\partial u_{j}},\left(\frac{\partial F(\psi ,m)}{\partial m}\right)\delta m\rangle }_{Q}.$$

Furthermore, if we consider a new state variable *v* belonging to the complex space V, given by:

(39) $$v={\left[{\left(\frac{\partial F(\psi ,m)}{\partial \psi_{j}}\right)}^{-1}\right]}^{†}\frac{\partial f(\psi ,m)}{\partial \psi_{j}},$$

where *v* is the first term of the inner product in Equation (38), we have the following equation of state:

(40) $${\left(\frac{\partial F(\psi ,m)}{\partial \psi_{j}}\right)}^{†}v=\frac{\partial f(\psi ,m)}{\partial \psi_{j}},$$

which is known as the adjoint-state equation [58,59], and therefore *v* is called the adjoint-state variable.

In summary, the calculation of the gradient of the κ-objective function through the state-adjoint method is given by:

(41) $$\nabla_{m}\phi_{\kappa }\left(m\right)=\frac{\partial \phi_{\kappa }\left(m\right)}{\partial m}=\frac{\partial f(\psi ,m)}{\partial m}-{\langle v,\frac{\partial F(\psi ,m)}{\partial m}\rangle }_{Q},$$

where the state-adjoint variable *v* is calculated from the state-adjoint equation in (40). In this way, for our problem we have:

(42) $$f\left(\psi_{s}\left(m,t\right),m\right)=-\sum_{r}\ln\left\{\exp_{\kappa }\left[-\beta_{\kappa }\;{\left(\Gamma_{s,r}\psi_{s}\left(m,t\right)-d_{s,r}\left(t\right)\right)}^{2}\right]\right\},$$

where $\phi_{\kappa }\left(m\right)=\sum_{s}f\left(\psi_{s}\left(m,t\right),m\right)$. In addition, for any model parameter *m*, let $\psi_{s}$ be a solution of the equation of state given in (32), that is, a physical realization. We obtain:

(43) $$F\left(\psi_{s}\left(t\right),m\right)=A\left(m,t\right)\psi_{s}\left(t\right)-g_{s}\left(t\right)=0$$

and

(44) $$f\left(\psi_{s}\left(t\right),m\right)=-\sum_{r}\ln\left\{\exp_{\kappa }\left[-\beta_{\kappa }\;{\left(\Gamma_{s,r}\psi_{s}\left(t\right)-d_{s,r}\left(t\right)\right)}^{2}\right]\right\}=f\left(\psi_{s}\left(t\right)\right).$$

Therefore, we obtain the following derivatives of the equation of state:

(45) $$\frac{\partial F(\psi_{s}\left(t\right),m)}{\partial m}=\frac{\partial A(m,t)}{\partial m}\psi_{s}\left(t\right)\;\mathrm{and}\;\frac{\partial F(\psi_{s}\left(t\right),m)}{\partial \psi_{s_{j}}}=A\left(m,t\right),$$

and the following for the κ-objective function:

(46) $$\frac{\partial f(\psi_{s}\left(t\right),m)}{\partial m}=0\;\mathrm{and}\;\frac{\partial f(\psi_{s}\left(t\right),m)}{\partial \psi_{s_{j}}}=\sum_{r}\frac{2\beta_{\kappa }\Gamma_{s,r}^{†}\left(\Gamma_{s,r}\psi_{s}\left(t\right)-d_{s,r}\left(t\right)\right)}{\sqrt{1+\kappa^{2}\beta_{\kappa }^{2}{\left(\Gamma_{s,r}\psi_{s}\left(t\right)-d_{s,r}\left(t\right)\right)}^{4}}}.$$

Thus, the gradient of the κ-objective function via the state-adjoint method is given by substituting the derivatives calculated in Equations (45) and (46) in Equations (40) and (41), as follows:

(47) $$\nabla_{m}\phi_{\kappa }\left(m\right)=-\sum_{s}\int_{0}^{T}{\langle v_{s}\left(\vec{x},t;\kappa \right),\frac{\partial^{2}\psi_{s}\left(\vec{x},t\right)}{\partial t^{2}}\rangle }_{\vec{x}}dt$$

with $v_{s}$ being the solution of the adjoint-wave equation given by

(48) $$m\left(\vec{x}\right)\frac{\partial^{2}v_{s}\left(\vec{x},t\right)}{\partial t^{2}}-\nabla^{2}v_{s}\left(\vec{x},t\right)=\sum_{r}\frac{2\beta_{\kappa }\Gamma_{s,r}^{†}\left(\Gamma_{s,r}\psi_{s}\left(t\right)-d_{s,r}\left(t\right)\right)}{\sqrt{1+\kappa^{2}\beta_{\kappa }^{2}{\left(\Gamma_{s,r}\psi_{s}\left(t\right)-d_{s,r}\left(t\right)\right)}^{4}}}$$

where $m\left(\vec{x}\right)=\frac{1}{c^{2}\left(\vec{x}\right)}$.

In search of a physical meaning for Equations (47) and (48), let us consider a new state variable given by $\lambda_{s}\left(\vec{x},t\right)=v_{s}\left(\vec{x},T-t\right)$. So, the latter equation becomes:

(49) $$m\left(\vec{x}\right)\frac{\partial^{2}\lambda_{s}\left(\vec{x},t\right)}{\partial t^{2}}-\nabla^{2}\lambda_{s}\left(\vec{x},t\right)=\sum_{r}\frac{2\beta_{\kappa }\Gamma_{s,r}^{†}\left(\Gamma_{s,r}\psi_{s}\left(T-t\right)-d_{s,r}\left(T-t\right)\right)}{\sqrt{1+\kappa^{2}\beta_{\kappa }^{2}{\left(\Gamma_{s,r}\psi_{s}\left(T-t\right)-d_{s,r}\left(T-t\right)\right)}^{4}}}.$$

We notice that the adjoint-state variable $\lambda_{s}$ is calculated in reverse time from Equation (49), i.e., starting the wave propagation from the final time *T* to the initial time 0. For this reason, this state-adjoint variable is commonly called the backpropagated wavefield, while Equation (49) is called the adjoint-wave equation, in which the right-hand term is named the adjoint source [59]. In this way, the κ-objective function gradient is calculated efficiently from the cross-correlation of the forward wavefield with the backpropagated wavefield.

In this context, computing the gradient via the state-adjoint method for each seismic source requires solving the wave equation only twice, first in the forward modeling and second in backpropagation modeling. We also point out that the robustness properties of the objective function discussed in Section 2 are indispensable in calculating the gradient. Indeed, the influence function (18) gives the adjoint source used in the inversion process. The particular classical case κ→0 provides the residual data as the adjoint source.

### 4.3. κ-Graph-Space Optimal Transport FWI

From a statistical point of view, non-Gaussian criteria are critical to handle noisy datasets in FWI analysis [9]. In this sense, we also consider the κ-Gaussian-based metric to deal with a challenging issue in FWI called cycle skipping [7]. Cycle skipping occurs when the initial model used in the FWI process is not kinematically accurate or lacks low-frequency contents in the analyzed dataset [34]. So, we consider the criterion based on κ-Gaussian statistics in the context of OT metric to mitigate the effects of non-Gaussian errors and cycle-skipping issues. However, let us remind the reader that the OT metric measures the distance between probability distributions, incompatible with a comparison between seismic signals, as discussed earlier. Thus, to work around this incompatibility, in this work we represent the non-normalized and oscillatory waveforms in the graph space [47]. Graphs are mathematical structures formed by ordered pairs of disjoint sets (V,E), where *V* denotes the so-called vertices and *E* represents an edge that connects paired vertices [60].

Hence, we discretize the waveforms d(t) as an ensemble of ordered pairs of the form $\left\{\left(t_{i},d_{i}\right)\in R^{2};i=1,2,\cdots ,N\right\}$ with $d_{i}=d\left(t_{i}\right)$. So, the graph-transformed representation of a discretized waveform $d=\{d_{i};i=1,2,\cdots ,N\}$ is defined as:

(50) $$\begin{array}{c} G:d\to G\left(d\right)=d^{G}\left(y,t\right)\\ \;\;R^{N}\to D\left(R^{2}\right), \end{array}$$

where G denotes the graph transformation, $d^{G}\left(y,t\right)$ is the graph-transformed waveform, and $D(R^{2})$ is a probability space on $R^{2}$. The graph-transformed waveform is defined as:

(51) $$d^{G}\left(y,t\right)=\frac{1}{N}\sum_{i=1}^{N}\delta \left(t-t_{i}\right)\delta \left(y-d_{i}\right),$$

where *y* is associated with the waveform amplitude. In this way, waveforms are represented by normalized and positive quantities.

However, in many contexts like FWI, one needs to calculate the derivative of waveforms on some occasions (as explained in the previous sections); the Dirac delta function is not differentiable. Due to this, we consider a smoothed graph transformation by representing Dirac functions using κ-Gaussian distributions (10). Thus, the κ-graph-transformed representation of a discretized waveform is given by [61]:

(52) $$\begin{array}{c} G_{\kappa }:d\;\to G_{\kappa }\left(d\right)=d^{G_{\kappa }}\left(y,t\right)\\ \;\;R^{N}\to C^{\infty }\left(R,R_{*}^{+}\right) \end{array}$$

with

(53) $$d^{G_{\kappa }}\left(y,t\right)=\frac{1}{Z_{\kappa }}\sum_{i=1}^{N}\exp_{\kappa }\left(-\beta_{\kappa }{\left(t-t_{i}\right)}^{2}\right)\exp_{\kappa }\left(-\beta_{\kappa }{\left(y-d_{i}\right)}^{2}\right),$$

where $C^{\infty }\left(R,R_{*}^{+}\right)$ represents a set of strictly positive and infinitely differentiable functions. In this context, the graph-space κ-OT objective function is defined as:

(54) $$\phi_{W_{\kappa }^{G_{\kappa }}}\left(m\right):=\sum_{s,r}C_{\kappa }\left(d_{s,r}^{mod}\left(m\right),d_{s,r}^{obs}\right),$$

where $d_{mod,i}^{G_{\kappa }}=\left(t_{i},d_{mod,i}\right)$ and $d_{obs,i}^{G_{\kappa }}=\left(t_{i},d_{obs,i}\right)$, and  $C_{\kappa }\left(d^{mod},d^{obs}\right)=W_{\kappa }^{G_{\kappa }}\left(d_{mod}^{G_{\kappa }},d_{obs}^{G_{\kappa }}\right)$ represents the κ-Wasserstein criterion applied to the graph-transformed seismic data. The κ-Wasserstein distance $W_{\kappa }^{G_{\kappa }}\left(d_{mod}^{G_{\kappa }},d_{obs}^{G_{\kappa }}\right)=W_{\kappa }^{G_{\kappa }}$ is then computed via the following minimization task:

(55) $$W_{\kappa }^{G_{\kappa }}=\underset{\sigma \in S(N)}{min}\;\;-\sum_{i=1}^{N_{t}}\ln\left\{\exp_{\kappa }\left[-\beta_{\kappa }{\left(t_{\sigma (i)}-t_{i})\right)}^{2}\right]\;\;\exp_{\kappa }\left[-\beta_{\kappa }{\left(d_{\sigma (i)}^{mod}-d_{i}^{obs})\right)}^{2}\right]\right\}.$$

where $N_{t}$ denotes the number of time samples for each waveform, while σ is the permutation solution for the linear sum assignment problem in (21) related with a transport map T, and S(N)={1,2,···,N} is an ensemble of permutations. For simplicity, we multiply the κ-Wasserstein distance (23) by the scalar *N* in the latter equation. Indeed, optimizing $W_{\kappa }$ is equivalent to optimizing the product $N\times W_{\kappa }$. Equation (55) represents the FWI objective function based on κ-OT, namely, the κ-GSOT-FWI, for short, in reference to κ-Graph-Space Optimal Transport FWI.

The gradient of the κ-GSOT-objective function (55), that is, the derivative of $W_{\kappa }^{G_{\kappa }}$ with respect to the model parameters, is given by:

(56) $$\nabla_{m}W_{\kappa }^{G_{\kappa }}\left(m\right)=\frac{\partial W_{\kappa }^{G_{\kappa }}\left(m\right)}{\partial m}=\sum_{s=1}^{N_{s}}\sum_{r=1}^{N_{r}}\sum_{i=1}^{N_{t}}J_{s,r,i}\left(m\right)U_{s,r,i}\left(m;\kappa \right),$$

in which

(57) $$U_{s,r,i}\left(m;\kappa \right)=\frac{2\beta_{\kappa }\left(d_{s,r,\sigma (i)}^{mod}\left(m\right)-d_{s,r,i}^{obs}\right)}{\sqrt{1+\kappa^{2}\beta_{\kappa }^{2}{\left(d_{s,r,\sigma (i)}^{mod}\left(m\right)-d_{s,r,i}^{obs}\right)}^{4}}}.$$

is the adjoint-source related with the κ-GSOT-FWI framework, while $N_{s}$, $N_{r}$ and $N_{t}$ represent the number of seismic sources, receivers, and time samples used in the acquisition of seismic data.

The statistical interpretation of the residual data (error) associated with the κ-Gaussian statistics is preserved in the κ-GSOT-FWI case. The critical difference is that in the approach without OT, the waveforms are compared sample by sample. In contrast, in the κ-GSOT-FWI approach, the waveforms are analyzed more completely, comparing each time sample of the observed data with all the time samples of the modeled data in according to an optimal assignment using the permutation solution σ.

**Figure 3:**
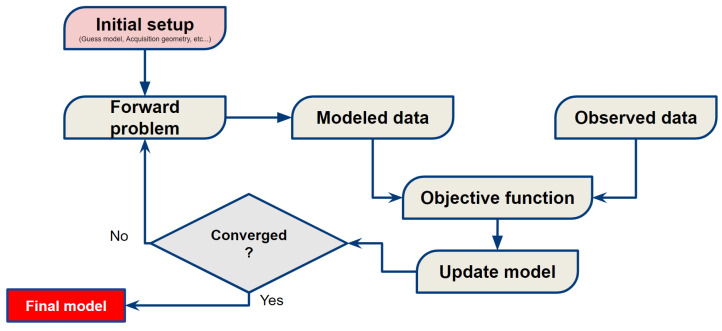
Flow chart of the full-waveform inversion (FWI) process.

Figure 3 shows a flow chart of the FWI algorithm, which is an iterative process, which means that model updates are computed concerning the previous model as described by Equation (24). The first step, called Initial Setup, consists of introducing the input variables, i.e., the initial model, the parameters of the seismic acquisition (the positions of the sources and receivers, the seismic source signature, acquisition time). After configuring and organizing all the input variables of the FWI algorithm, modeled wavefields are obtained in the forward problem through the numerical solution of the wave Equation (28) by employing the finite difference method [62]. Then, a sampling operation ($\Gamma_{s,r}$) is carried out from the modeled wavefields $\psi_{s}$ to obtain the modeled data ($d_{s,r}^{mod}=\Gamma_{s,r}\psi_{s}$), extracting the wavefields in the positions of the seismic acquisition receivers. After, the objective function gradient is obtained through the adjoint-state method described in Section 4.2 and used to update the model following Equation (24). Finally, the FWI algorithm checks whether the optimization process reached the pre-defined stopping criteria (which, in our case, was the maximum number of iterations equal to 50). As long as the criteria are not met, the cycle is repeated. If so, the iterative process is interrupted, and the resulting model is the one that minimizes the difference between modeled and observed data.

## 5. Numerical Experiments

**Figure 4:**
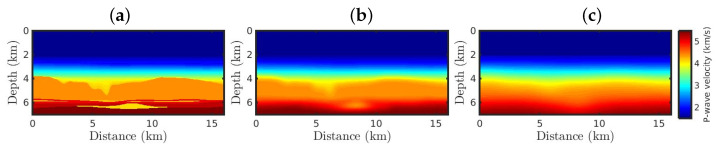
(**a**) Chalda model representing the Brazilian pre-salt oil region, used as the true model. Initial models used in the (**b**) first and (**c**) second scenarios.

To demonstrate how the κ-GSOT-FWI deals with non-Gaussian noise and cycle-skipping issues, we carried out numerical examples involving a 2D acoustic time-domain FWI to estimate a P-wave velocity model in a typical Brazilian pre-salt oil region. Such an Earth model, namely, Chalda, represents a region with approximate dimensions 16 by 7 km in lateral distance and depth, respectively, as depicted in Figure 4a. Our problem has 720,484 unknown variables because we discretize the Chalda model in a regular grid with 12.5 m spacing, generating 562 and 1282 grid cells in the vertical and horizontal directions, respectively.

In all numerical experiments, we consider a seismic survey comprising 161 seismic sources equally spaced every 75 m at 12.5 m in-depth. We employ a Ricker wavelet as a seismic source, which is mathematically described by: $f\left(t\right)=\left(1-2\pi^{2}\mu_{p}^{2}t^{2}\right)\exp\left(-\pi^{2}\mu_{p}^{2}t^{2}\right)$, in which $\mu_{p}$ represents the peak frequency (maximum energy in the spectrum of frequencies). Moreover, to simulate a sparse node acquisition, named the ocean bottom nodes survey, we take into account 21 receivers implanted on the ocean floor at 400 m intervals. We consider the Chalda model depicted in Figure 4a as a benchmark (or true model). Thus, we generate a seismic dataset by considering the true model, the acquisition geometry, and the finite difference method with second and eighth order approximations for time and space. In order to simulate an infinite medium, we implement the perfectly matched layer [63] absorbing boundaries for spatial discretization. We consider 7 s as the seismic acquisition time at a sampling rate of 2 ms. In addition, to simulate a realistic case, we also employ a high-pass filter on the seismic dataset to remove energy less than 2.5 Hz.

In the FWI experiments, we consider two scenarios involving different initial models to confirm the significance of our proposal. In the first one, we consider an initial model similar to the true model, which is depicted in Figure 4b. We produce such a velocity model by weakly smoothing the true model by applying a Gaussian filter with a standard deviation of 250 m. This scenario’s idea is to simulate a seismic imaging process starting from a kinetically accurate model. We call this model the Good Model. In contrast, we produce the second initial model, referring to the second scenario, by applying a more severe Gaussian filter with a standard deviation of 750 m. We call this model the Bad Model. We notice that the Bad Model lacks the main structures of the true model, particularly in the pre-salt oil region, as depicted in Figure 4c. Since the Bad Model is kinematically inaccurate, it generates cycle-skipped data [34].

For each initial-model scenario, we conduct time-domain FWI by applying the classical FWI based on Gaussian statistics, and the κ-GSOT objective function (55) in the classical limit κ→0 and for κ=0.1, 0.3, 0.5 and 0.6. We consider 50 FWI iterations in all numerical experiments. To evade the so-called inversion crime, we perform the forward modeling using a different algorithm than the one used to generate the observed dataset. In this regard, our algorithm solves the forward problem using a finite difference scheme with second and fourth order approximations for time and space in a regular grid with 25 m spacing. In addition, we consider two different circumstances concerning the type of noise in the seismic dataset. First, we consider a dataset contaminated by Gaussian noise with a signal-to-noise ratio (SNR) of 20. In contrast, in the second circumstance, we consider a non-Gaussian noise from which the dataset is polluted by Gaussian noise with an SNR of 20 and a collection of spikes (erratic data or outliers) with different amplitudes. In this regard, 10% of the time samples were contaminated by outliers, where locations were randomly drawn. The spikes have intensities that range from 5P to 15P multiplied by the original waveform amplitude, where *P* is a standard normal random variable.

We perform our numerical simulations using a computer hosting a quad-core processor at 3.50 GHz and 256 GB RAM. Each FWI iteration takes approximately 6 min; 71.4% is associated with calculating the gradient of the objective function using the state-adjoint method described in Section 4.2, 26.7% is related to the forward modeling process (i.e., in generating the modeled data by numerically solving Equation (28)), 1.3% of this time is dedicated to solving the combinatorial optimization problem in (55), and 0.6% is spent on the rest of the algorithm in I/O initialization and initial set-ups loading.

**Figure 5:**
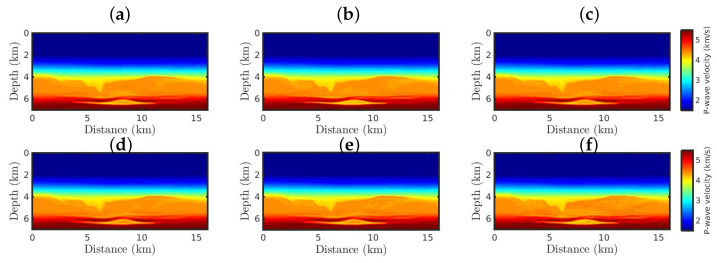
The resulting models starting from the Good Model for the Gaussian noise case, by employing the (**a**) classical FWI approach, and the κ-GSOT-FWI framework with (**b**) κ→0, (**c**) κ=0.1, (**d**) κ=0.3, (**e**) κ=0.5, and (**f**) κ=0.6.

**Figure 6:**
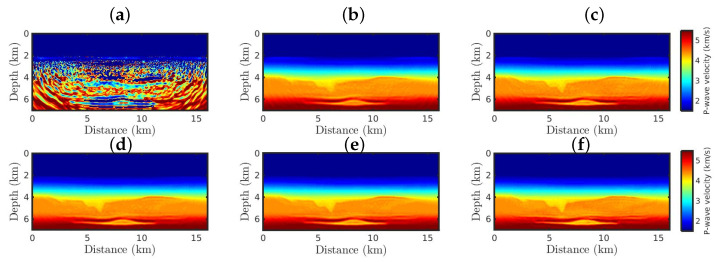
The resulting models starting from the Good Model for the non-Gaussian noise case, by employing the (**a**) classical FWI approach, and the κ-GSOT-FWI framework with (**b**) κ→0, (**c**) κ=0.1, (**d**) κ=0.3, (**e**) κ=0.5, and (**f**) κ=0.6.

Figure 5 and Figure 6 show the FWI resulting P-wave models starting from the Good Model for the Gaussian and non-Gaussian noise cases, respectively. From a visual inspection, when only Gaussian noise is considered, all resulting models are satisfactory (Figure 5) since they are very similar to the true model (Figure 4a), regardless of the κ-value. Such successful results are due to the weak Gaussian noise in the observed data simultaneously with a kinetically accurate initial model (Figure 4b).

Furthermore, we quantitatively compare our FWI resulting models with the true model by employing Pearson’s correlation coefficient (R) and the normalized root-mean-square (NRMS), defined as

(58) $$R=\frac{\mathrm{cov}\left(c^{true},c^{inv}\right)}{\mathrm{std}\left(c^{true}\right)\;\mathrm{std}\left(c^{inv}\right)}\;\mathrm{and}\;\mathrm{NRMS}={\left[\frac{\sum_{i}{\left(c_{i}^{true}-c_{i}^{inv}\right)}^{2}}{\sum_{i}{\left(c_{i}^{true}\right)}^{2}}\right]}^{1/2},$$

where $c^{true}$ and $c^{inv}$ are the true and the resulting models, while cov(·) and std(·) denote covariance and standard deviation, respectively. The R-value ranges from −1 to 1, with −1 representing, in this context, a wrong resulting model, while 1 represents a perfect resulting model. The NRMS-value range from 0 (perfect resulting model) to *∞* (wrong resulting model).

Table 1 summarizes the comparative metrics between the true model and the P-wave velocity models resulting from the first scenario by analyzing data contaminated only by Gaussian noise. In this table, we can see that all resulting models have a low error and are strongly correlated with the true model (R≥0.8, following the strength-scale suggested by ref. [64]).

However, when non-Gaussian noise is considered, the classical approach fails as expected (Figure 6a). Such a wrong model is due to the classical approach being based on Gaussian statistics and sensitive to cycle-skipping issues. Figure 6b shows the resulting model from the classical GSOT-FWI, which is also based on Gaussian statistics. However, the Wasserstein metric was able to mitigate the effects of the outliers, building a satisfactory model. Nevertheless, as the κ-value increases (which means a more significant deviation from Gaussian behaviors), the κ-GSOT-FWI models present a better resolution (Figure 6c–f), especially in the deeper regions of the analyzed area. Although all κ-GSOT-FWI models are strongly correlated with the true model, the case κ=0.6 has a higher Pearson’s coefficient and a smaller NRMS error, as summarized in Table 2.

**Figure 7:**
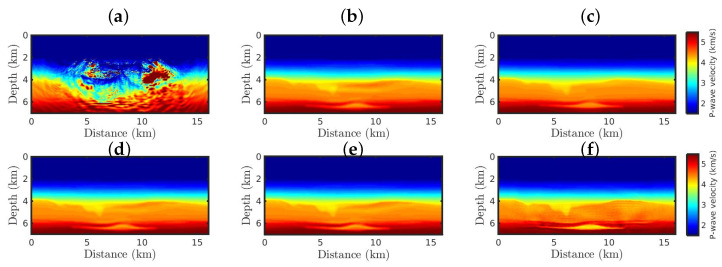
The resulting models starting from the Bad Model for the Gaussian noise case, by employing the (**a**) classical FWI approach, and the κ-GSOT-FWI framework with (**b**) κ→0, (**c**) κ=0.1, (**d**) κ=0.3, (**e**) κ=0.5, and (**f**) κ=0.6.

**Figure 8:**
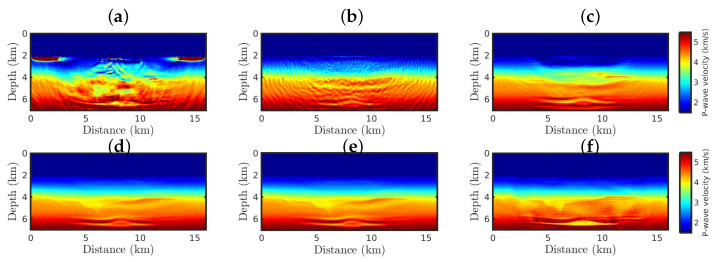
The resulting models starting from the Bad Model for the non-Gaussian noise case, by employing the (**a**) classical FWI approach, and the κ-GSOT-FWI framework with (**b**) κ→0, (**c**) κ=0.1, (**d**) κ=0.3, (**e**) κ=0.5, and (**f**) κ=0.6.

Figure 7 and Figure 8 show the FWI resulting P-wave models starting from the Bad Model for the Gaussian and non-Gaussian noise cases, respectively. From a visual inspection, it is noticeable that the classical FWI approach fails when the initial model is kinetically inaccurate, regardless of whether the data are polluted by Gaussian or non-Gaussian noise, as depicted in Figure 7a and Figure 8a. In contrast, the FWI based on the κ-GSOT approach generates satisfactory models when Gaussian noise is considered, regardless of the κ-value (Figure 7b–f). Again, as the κ-value increases, the resulting models (Figure 7) are closer to the true model (Figure 4a), as endorsed by the statistical metrics summarized in Table 3.

Finally, in the second scenario with non-Gaussian noise, the resulting models are drastically affected by the outliers and poverty from the initial model, as depicted in Figure 8. However, when the κ-GSOT-based objective function is applied, the large geological structures of the true model are reconstructed regardless of the κ-value. However, the case κ=0.6 reveals a P-wave velocity model (Figure 8f) that is quite accurate and comparable to the true model (Figure 4a). Likewise, the case κ=0.6 generated a model closer to the true model, as summarized in Table 4. Indeed, in all numerical tests, the κ-GSOT-FWI for κ=0.6 generated accurate velocity models, leading to accurate parameter estimations.

**Figure 9:**
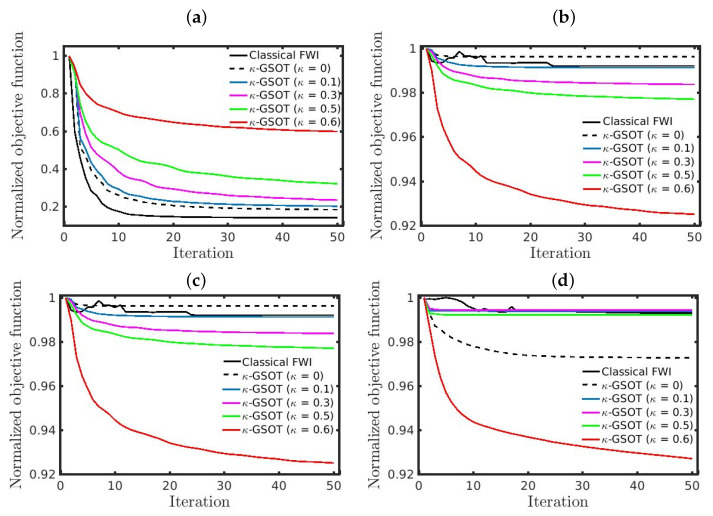
Convergence curves for the first scenario with (**a**) Gaussian noise, (**b**) non-Gaussian noise, and for the second scenario with (**c**) Gaussian noise, (**d**) non-Gaussian noise.

Figure 9 shows the normalized κ-GSOT-objective function decay for all numerical tests, in which panels (a) and (b) refer to the first scenario, while panels (c) and (d) correspond to the second scenario. In this regard, the left column refers to the case in which Gaussian noise is considered, and the right column is the non-Gaussian noise case. The convergence curve of the classical objective is represented by the solid black line in Figure 9. We notice that the classical objective function monotonically decays only in the most straightforward situation, where the initial model is the Good Model, and the data is contaminated by Gaussian noise (Figure 9a). In this case, the classical approach is the most indicated because the convergence rate is higher than our proposal, in addition to generating more accurate models (as summarized in Table 1). In cases where the noise is non-Gaussian or when the inversion process starts from the Bad Model, our proposal with κ=0.6 exhibits a higher objective function decay rate (see red curves in Figure 9b–d), reconstructing P-wave velocity models closer to the true model, as summarized in Table 2, Table 3 and Table 4.

## 6. Final Remarks

In this work, we have examined the portability of the objective function based on the graph-space optimal transport and Kaniadakis κ-Gaussian statistics in the FWI context. In particular, we have analyzed the robustness of our proposal in mitigating two critical problems in seismic imaging via FWI, which are associated with cycle-skipping issues and the non-Gaussian nature of the errors. We have set up an objective function by employing the probabilistic maximum likelihood method for computing the most probable state using a κ-Gaussian distribution. Furthermore, we have formulated the FWI in a relaxed version of the optimal transport problem, known as the Kantorovich–Rubinstein metric or Wasserstein distance. So, we have considered the graph of the seismic data rather than the original data because the optimal transport framework is predicated on the idea that the compared entities adhere to the probability axioms. We named our proposal the κ-Graph-Space Optimal Transport FWI (or κ-GSOT-FWI, for short).

The Brazilian pre-salt case study disclosed how the κ-GSOT-FWI could be employed to deal with flawed initial models and non-Gaussian noise. The findings have demonstrated that the classical approach is ineffective in producing accurate physical models when the initial model is crude or if the observed waveforms are contaminated by non-Gaussian errors. However, when the initial model is kinetically precise and the data well-behaved, the classical approach is the best alternative in terms of computational cost. The results also revealed that the κ-GSOT-FWI lessens the impact of phase ambiguity and non-Gaussian errors on the waveform inversion, demonstrating that our proposal is a powerful way to deal with non-linear inverse problems related to wave propagation. Moreover, we notice that the κ-GSOT-FWI produces more accurate models than those produced by classical approaches, leading to a notable improvement in objective function convergence. Additionally, our numerical experiments demonstrated that a more significant deviation from a Gaussian behavior (which in our applications was typified by the κ=0.6 case) results in a more authentic P-wave velocity model. However, our proposal depends on the choice of a hyperparameter, which demands special investigations on how to obtain it in a real setting application. This issue should be examined in future applications.

From a practical point of view, extensive and arduous data processing is required to engineer a good initial model to alleviate phase-ambiguity issues and eliminate erratic data points. In this context, the κ-GSOT-FWI decreases the requirement of human subjectivity, which is appealing for automated techniques to analyze, for instance, recent big datasets. Thus, the κ-OT-based approach has enormous potential for dealing with modern data-centric problems. As a perspective, we intend to test our proposed methodology to analyze field data and evaluate its robustness from several initial conditions. Finally, we underline how readily our concept may be applied to a wide variety of inverse problems, ranging from estimating critical exponents of power-law distributions to modern artificial intelligence applications.

## Figures and Tables

**Table 1:** The comparative metrics between the true model and the resulting models, depicted in Figure 1, from the first scenario in the Gaussian noise case. R represents the Pearson’s correlation coefficient, while NRMS represents the normalized root-mean-square.

Strategy	κ	NRMS	R
Classical FWI	-	0.0256	0.9982
	0.0	0.0272	0.9979
	0.1	0.0278	0.9979
κ-GSOT-FWI	0.3	0.0288	0.9977
	0.5	0.0293	0.9976
	0.6	0.0277	0.9979

**Table 2:** The comparative metrics between the true model and the resulting models, depicted in Figure 6, from the first scenario in the non-Gaussian noise case. R represents the Pearson’s correlation coefficient, while NRMS represents the normalized root-mean-square.

Strategy	κ	NRMS	R
Classical FWI	-	0.3009	0.7627
	0.0	0.0306	0.9974
	0.1	0.0296	0.9976
κ-GSOT-FWI	0.3	0.0293	0.9976
	0.5	0.0293	0.9976
	0.6	0.0277	0.9979

**Table 3:** The comparative metrics between the true model and the resulting models, depicted in Figure 7, from the second scenario in the Gaussian noise case. R represents the Pearson’s correlation coefficient, while NRMS represents the normalized root-mean-square.

Strategy	κ	NRMS	R
Classical FWI	-	0.2947	0.7982
	0.0	0.0362	0.9964
	0.1	0.0333	0.9970
κ-GSOT-FWI	0.3	0.0363	0.9964
	0.5	0.0372	0.9962
	0.6	0.0341	0.9968

**Table 4:** The comparative metrics between the true model and the resulting models, depicted in Figure 8, from the second scenario in the non-Gaussian noise case. R represents the Pearson’s correlation coefficient, while NRMS represents the normalized root-mean-square.

Strategy	κ	NRMS	R
Classical FWI	-	0.2715	0.7192
	0.0	0.0610	0.9899
	0.1	0.0673	0.9874
κ-GSOT-FWI	0.3	0.0564	0.9913
	0.5	0.0578	0.9908
	0.6	0.0509	0.9928

## Data Availability

Not applicable.

## Abbreviations

The following abbreviations are used in this manuscript:

FWI	Full-Waveform Inversion
GSOT	Graph-Space Optimal Transport
OT	Optimal Transport
MLE	Maximum Likelihood Estimation
NRMS	Normalized Root-Mean-Square
